# The Effects of Transcutaneous Tibial Nerve Stimulation on Female Sexual Function in Multiple Sclerosis Patients: A Narrative Review

**DOI:** 10.7759/cureus.77306

**Published:** 2025-01-12

**Authors:** Athanasios Zachariou, Dimitrios Baltogiannis, Athanasios Zikopoulos, Vaia Sapouna, Ioannis Giannakis, Aris Kaltsas, Vladimir Kojovic, Fotios Dimitriadis, Atsushi Takenaka, Nikolaos Sofikitis

**Affiliations:** 1 Urology, University of Ioannina, Ioannina, GRC; 2 Obstetrics and Gynecology, Royal Cornwall Hospital, Truro, GBR; 3 Physiotherapy, Clinical Exercise Physiology and Rehabilitation Laboratory, University of Thessaly, Lamia, GRC; 4 Physical Therapy, Physical Medicine and Rehabilitation Centre Kentavros, Volos, GRC; 5 Urology, Attikon University Hospital, School of Medicine, National and Kapodistrian University of Athens, Athens, GRC; 6 Urology, University of Belgrade, Belgrade, SRB; 7 Urology, Aristotle University of Thessaloniki, Thessaloniki, GRC; 8 Surgery, School of Medicine, Tottori University, Yonago, JPN

**Keywords:** female sexual dysfunction, multiple sclerosis, neuromodulation therapy, pelvic floor muscle training, transcutaneous tibial nerve stimulation

## Abstract

Female sexual dysfunction (FSD) is prevalent among women with multiple sclerosis (MS), leading to significant impairments in quality of life. Despite various available treatments, current approaches often fail to address the complex, multifactorial nature of FSD in this population. Transcutaneous tibial nerve stimulation (TTNS) has recently gained attention as a non-invasive neuromodulation therapy that has demonstrated potential benefits for neurogenic bladder dysfunction, with preliminary evidence suggesting overlapping improvements in sexual function. This narrative review synthesizes current evidence on the use of TTNS for managing FSD in women with MS. A comprehensive literature search of Medline, Web of Science, and Scopus was conducted, focusing on studies that explored TTNS interventions and outcomes in MS-related FSD. The findings indicate that TTNS likely modulates pelvic neural pathways, enhances genital blood flow, and improves key sexual function domains, including arousal, lubrication, and orgasm. Clinical studies using validated assessment tools, such as the Female Sexual Function Index (FSFI), have shown significant improvements in sexual outcomes, particularly when TTNS is combined with pelvic floor muscle training (PFMT). Experimental models further support the plausibility of these findings, linking TTNS to increased pelvic perfusion and more balanced neurogenic regulation of sexual response. Although initial evidence suggests that TTNS is safe, effective, and well-tolerated, limitations include small sample sizes, short follow-up periods, and variability in treatment protocols. Future research should focus on larger controlled trials, long-term follow-up studies, and standardized intervention parameters to optimize the application of TTNS for addressing FSD in women with MS.

## Introduction and background

Multiple sclerosis (MS) is a chronic autoimmune disease that damages the central nervous system (CNS), specifically targeting the myelin sheath, a protective layer around nerve fibers, resulting in disrupted communication between the brain and other parts of the body. It is among the most prevalent demyelinating neurodegenerative conditions, with an estimated 2.3 million patients globally [1]. This condition causes symptoms such as fatigue, mobility issues, and neurological impairments, and its progression is influenced by both genetic and environmental factors. The immunological factors of MS highlight the critical need to explore the complex interactions between the immune system and the CNS within the disease’s framework [2]. The disease is significantly more prevalent in women, with a female-to-male ratio of approximately 3:1. Female sexual dysfunction (FSD), defined as persistent problems with sexual response, desire, orgasm, or pain, is a particularly relevant issue for women with MS, especially since the disease often begins during their reproductive years [3]. The interplay between MS and FSD is driven by a combination of neurological, physical, and psychosocial factors, including impaired nerve signaling, spasticity, and emotional challenges.

Over time, many individuals with MS experience spasticity, sensory impairments, vision problems like diplopia, bladder and bowel dysfunction, fatigue, and frequently, sexual dysfunction (SD). MS is strongly associated with long-term urogenital issues, with studies estimating that 40-80% of women with MS encounter some form of SD during their lives [4]. In general, reduced sexual activity, reduced sexual satisfaction, difficulties related to reaching orgasm, and reduced vaginal lubrication have been reported as the main issues for women. For healthcare providers, addressing these challenges is complicated by a lack of awareness regarding effective treatments and the stigma associated with discussing sexual health.

Traditional treatment approaches, including psychoeducational and behavioral interventions, pharmacological approaches, and interventional treatments, may not always provide sufficient relief. The ability to specify these findings to a specific population of people with MS is limited by several factors, including small sample sizes, the inclusion of perimenopausal women, variability in levels of disability, and insufficient data on psychological comorbidities [4-6]. Additional constraints include the use of medications such as antidepressants and treatments for spasticity, the absence of long-term follow-up, and reliance on self-reported questionnaires. These questionnaires may either overstate the severity of SD symptoms due to emotional language or underreport them due to the stigma participants may feel in disclosing such issues [4,7]. 

Transcutaneous tibial nerve stimulation (TTNS) is a non-invasive neuromodulation technique that has been proposed for the treatment of neurogenic detrusor overactivity (NDO) in MS patients with promising results [8]. This method involves the application of mild electrical impulses to the tibial nerve using surface electrodes, which can be self-administered. While the exact mechanism behind nerve stimulation remains unclear, the anticipated theory for tibial nerve stimulation (TNS) is based on the presence of L4-S3 fibers, which originate from the same spinal segments of the parasympathetic nervous system as those associated with bladder function (L5-S3) [9]. The mechanism of action for TTNS is thought to parallel that of sacral nerve stimulation. It involves the modulation and reorganization of spinal reflexes and enhanced afferent input to the micturition center via the sacral nerve roots, ultimately restoring the balance between excitatory and inhibitory elements of bladder control [10,11]. 

TTNS is performed by applying surface electrodes to the skin, allowing individuals to self-administer treatment that targets the tibial nerve, a mixed nerve containing L5-S3 fibers. This technique has been associated with relief from urinary symptoms such as incontinence, urgency, frequency, and nocturia while addressing FSD. However, variability in treatment protocols limits its clinical applicability and highlights the need for standardization in future research [12]. 

Treatment for FSD in the context of MS requires a holistic approach. Addressing physical symptoms, such as spasticity and bladder dysfunction, is essential. Medications like antispasmodics and pelvic floor physical therapy can alleviate some of these issues. Psychological interventions, including counseling and cognitive-behavioral therapy, are equally important. These therapies help women navigate emotional barriers and improve communication with their partners [4,6]. This review aims to provide a comprehensive analysis of the role of TTNS in managing FSD in MS patients, synthesizing current literature to evaluate patient outcomes, techniques, and its overall impact on sexual function in women suffering from MS.

## Review

Neurophysiological mechanisms underlying sexual function 

Sexual function is an intricate process driven by the interplay between neurological, vascular, and endocrine systems, influenced further by psychological, social, economic, and cultural factors. The orchestration of sexual behavior and function relies on multiple brain and spinal cord structures, notably the limbic system, cerebral cortex, brainstem, and S2-S4 spinal cord segments. These regions coordinate key phases of the human sexual response cycle, including desire, arousal, and orgasm, which are deeply intertwined with cultural and ethical norms [13,14].

At the neurobiological level, sexual desire and pleasure are regulated by a complex interaction of the opioid-endocannabinoid system and the dopaminergic network, both integral to the brain's reward pathways. Neurons in the substantia nigra pars compacta and the ventral tegmental area of the midbrain play a crucial role in motivating sexual behavior and partner selection [15].

Specific limbic structures such as the amygdala, hypothalamus, hippocampus, and septal nuclei are essential for processing emotions, genital sensations, and motivational aspects of sexual activity. Higher-order brain regions like the prefrontal cortex and cingulate cortex regulate sexual impulses, while the insula integrates awareness of genital sensations during arousal [16].

The spinal cord, particularly the S2-S4 segments, is pivotal in coordinating genital function and sexual responses in both males and females. Efferent signals from the sacral parasympathetic nucleus travel via the pudendal nerve, which branches into the perineal and dorsal nerves, providing motor and sensory innervation to genital structures. In women, these neural pathways are essential for clitoral engorgement, vaginal lubrication, and genital sensation. Reflexive lubrication, a hallmark of the arousal phase in the female sexual response, is mediated by parasympathetic pathways originating from the sacral segments, while psychogenic arousal, involving increased blood flow to the clitoris and vaginal walls, is regulated by the thoracolumbar sympathetic system [17,18]. Disruption in these pathways, as seen in neurological disorders like MS, often leads to FSD. Reduced genital sensation, impaired lubrication, and difficulty achieving orgasm are common sequelae of such neural damage. The integration of neural signals with vascular responses is crucial for maintaining sexual function, and any dysregulation in this interplay can compromise sexual health. Understanding these mechanisms is essential for developing effective interventions, such as TTNS, which may modulate the sacral spinal reflexes involved in both bladder and sexual function [19,20].

A thorough understanding of these neurophysiological mechanisms is essential for developing effective treatments for SD, especially in individuals with spinal cord injuries (SCI) or neurological disorders, where these pathways are often disrupted.

MS and female sexual function 

Studies consistently show a significantly higher prevalence of FSD among women with MS compared to the general population, posing significant challenges to their overall well-being. A meta-analysis revealed that MS increases the risk of FSD by 1.87 times, with approximately 71% of women with MS experiencing some form of SD [21]. Factors such as disease duration, physical disability, and age contribute to this heightened risk. Women over 40 with MS are particularly vulnerable to FSD, showing significantly lower scores in all domains of sexual function, including desire, arousal, and lubrication [22]. 

The causes of FSD in MS are complex and continue to be a topic of research and debate. Neurological impairments associated with MS, such as demyelination and damage to nerve pathways, are central to the development of FSD. For instance, lesions in specific brain areas, such as the temporal and occipital regions, have been linked to sexual difficulties. Primary SD in MS directly results from neurological damage caused by demyelinating lesions. These lesions impair female sexual function by reducing genital sensation, diminishing sexual desire, arousal, and affecting the ability to achieve orgasm [23]. Secondary SD arises indirectly from MS-related physical symptoms, including spasticity, pain, fatigue, and bladder or bowel dysfunction, which interfere with sexual responsiveness. Fatigue, a common symptom of MS, exacerbates sexual difficulties in both men and women, while bladder dysfunction often serves as an indicator of underlying neurological damage and contributes to sexual issues. Neuropathic pain, which affects 60-70% of individuals with MS, combined with physical limitations like reduced mobility and spasticity, further hinders sexual activity [24].

Tertiary SD is shaped by psychosocial factors such as mood disorders and cognitive impairments, which diminish sexual satisfaction and create tension in relationships. Depression, experienced by 27-54% of individuals with MS, is closely linked to FSD, alongside anxiety and persistent fatigue. Research suggests that the progression of depression and fatigue correlates with a higher likelihood of worsening SD [25]. Studies suggest that mood disturbances, combined with a lack of body confidence and self-image issues, significantly affect sexual function and satisfaction. Cognitive impairments, commonly associated with MS, also play a significant role, as individuals with cognitive difficulties often report higher levels of SD and reduced social engagement [25].

Neurological pathway alterations can impact both sexual function and detrusor function. While the exact relationship between detrusor overactivity (DO) and SD has not been fully clarified, it is hypothesized that detrusor function impairment resulting from MS could serve as a meaningful indicator of severe neurological disability and associated SD [26,27]. Furthermore, according to Fragala et al, the results of urodynamic evaluation of neurogenic bladder in MS patients are associated with SD [24]. The incidence of maximal detrusor pressure during involuntary detrusor contraction (PdetmaxIDC) >20 cm H₂O, maximal cystometric capacity (MCC) <135 mL, and compliance <3 mL/cm H₂O may significantly predict the presence of moderate erectile dysfunction (ED). Concerning FSD, there was a positive relationship between MCC and the FSFI domains of arousal, lubrication, and pain.

Additionally, socioeconomic factors, including employment status, financial stability, and education level, are associated with SD in MS patients. FSD can emerge at various stages of the disease, often appearing early in its progression and becoming increasingly common as the condition advances. This interplay of neurological, physical, and psychosocial factors highlights the multifaceted nature of FSD in MS, underscoring the need for comprehensive management strategies tailored to address the diverse challenges faced by these patients [28].

FSD in women with MS is not solely a consequence of the disease's physical symptoms. The stigma surrounding SD, coupled with communication barriers between patients and healthcare providers, often prevents women from seeking or receiving appropriate care [29]. Moreover, the side effects of MS treatments, such as fatigue-inducing medications or those affecting libido, contribute to secondary SD. For instance, drugs like beta interferons and antidepressants are known to impact sexual desire and performance negatively.

Diagnosing FSD in women with MS is challenging due to the multifactorial nature of the dysfunction. Healthcare providers often overlook or underprioritize sexual health during consultations. Many patients, in turn, hesitate to discuss their sexual concerns due to embarrassment or cultural stigma [30]. The use of self-reported questionnaires, while helpful, sometimes leads to underestimation or overestimation of symptoms, further complicating diagnosis.

Methods 

A comprehensive literature search was performed across three major databases: Medline via PubMed, Web of Science, and Scopus. Targeted keywords were utilized, including terms such as "multiple sclerosis," "female sexual dysfunction," "transcutaneous tibial nerve stimulation," "overactive bladder (OAB)," and "neurogenic detrusor overactivity." To ensure thoroughness, reference lists of relevant articles were also carefully examined, emphasizing a robust exploration of the existing body of literature.

The inclusion criteria focused on studies regarding adult females with SD associated with MS. Particular attention was given to studies that provided thorough descriptions of detailed rehabilitation interventions. Eligible research designs included prospective and retrospective studies as well as randomized controlled trials (RCTs), with a preference for those published in English to maintain consistency in data analysis and interpretation.

To refine the scope of the review, specific exclusion criteria were applied. Studies focusing on neurological conditions other than MS were omitted to maintain the focus on the exclusive issues of MS-related SD. Additionally, studies that did not explicitly address sexual complications were omitted. To ensure the accuracy and reliability of findings, duplicate studies were systematically identified and excluded.

TTNS: mechanisms and applications 

TNS is an alternative treatment for OAB or NDO in patients who do not respond to conservative therapies. Currently, three TNS methods are commonly utilized: percutaneous tibial nerve stimulation (PTNS), implantable tibial nerve stimulation (ITNS), and TTNS. During PTNS, a needle electrode is inserted near the medial malleolus, which may lead to complications such as bleeding, infection, and discomfort. Similarly, ITNS involves implanting a stimulator into the tibial nerve, potentially causing side effects comparable to those seen with PTNS. Both PTNS and ITNS require administration by trained medical professionals.

**Figure 1:**
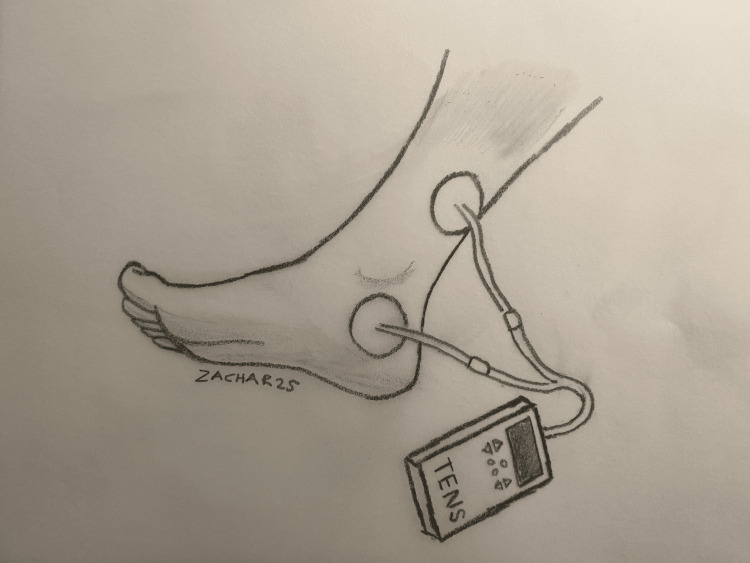
Correct placement of electrodes for transcutaneous electrical nerve stimulation: demonstration of electrode positioning and functional confirmation via plantar flexion of the great toe Image Credits: Authors

Various compact devices are available for TTNS, most of which utilize two electrodes. One electrode is positioned below the left medial malleolus, while the other is placed approximately 5 cm proximal to it. These electrodes are connected to a handheld transcutaneous electrical nerve stimulation (TENS) device, which delivers mild electrical impulses to the electrode pads. The correct placement of the electrodes is verified when stimulation produces plantar flexion of the great toe. The usual settings are a pulse duration of 200 μsec, a frequency of 10 Hz, and an intensity of 10-20 mA adjusted to the patient’s comfort level. Once the electrode placement is confirmed, the stimulation amplitude is reduced to just below the sensory threshold to ensure patient comfort [31]. Recent studies suggest following a 12-week treatment protocol, with three to five sessions scheduled per week, each lasting 20-30 minutes [10,32]. To visually demonstrate the setup and electrode placement for effective stimulation using the TENS device, Figure 1 has been provided.

The mechanisms underlying posterior TNS remain poorly understood, yet some insights suggest potential pathways of action. The tibial nerve interfaces with the spinal cord at the sacral roots, which are integral to pelvic organ innervation. This anatomical connection has led to the hypothesis that PTNS modulates afferent and efferent signals related to bladder function via the sacral plexus, employing retrograde afferent stimulation [33]. Another proposed mechanism involves posterior TNS enhancing pelvic blood flow, which may contribute to its therapeutic effects [34].

Clinical studies have revealed notable improvements in sexual functioning among women undergoing posterior TNS for lower urinary tract dysfunction. These benefits include enhanced arousal, desire, lubrication, and orgasmic ease, suggesting a broader impact of posterior TNS beyond bladder control [35,36]. Since bladder dysfunction is known to negatively affect sexual function, it is plausible that addressing bladder issues may indirectly restore aspects of sexual health [37,38]. However, the observation that improvements in sexual function are not consistently correlated with enhanced bladder performance implies that posterior TNS may exert direct effects on sexual function independent of bladder-related mechanisms.

Further evidence of posterior TNS's potential benefits comes from its application in women treated for chronic pelvic pain, where improved sexual functioning has also been reported [39]. Nonetheless, these outcomes may be partially attributable to secondary effects, such as alleviation of pain or bladder dysfunction, rather than a direct influence on sexual function. The lack of definitive separation between primary and secondary effects, as well as the limited exploration of these findings, underscores the need for more rigorous investigation into the specific mechanisms and broader therapeutic potential of posterior TNS.

TTNS devices should not be used in patients with cardiac pacemakers, defibrillators, electronic implants, or metal implants near the stimulation site, as they may cause interference or potential burns. In epileptic patients, further discussion is needed with the attending neurologist. Additionally, treatment is avoided in individuals with ankle joint issues or dermatological conditions at the intended electrode placement areas [10]. Patients rarely report side effects. However, some may experience mild cramping or discomfort along the inner part of the lower leg and foot or slight skin irritation at the electrode pad sites [40]. If a skin reaction occurs, treatment should be discontinued immediately, and a healthcare professional should be consulted. Additionally, if any skin irritation is noticed after a single session, it is essential to pause the treatment and seek medical advice. For optimal comfort, it is recommended that patients remain seated in a relaxed position throughout the 30-minute treatment session.

Experimental insights into TNS for FSD 

Initial experimental studies evaluated the effects of TNS on vaginal blood perfusion (VBP) in an anesthetized rat model, exploring its potential as a treatment for FSD, particularly for genital arousal issues. Zimmerman et al. studied 16 female rats that were surgically implanted with tibial nerve electrodes and underwent continuous or intermittent electrical stimulation at frequencies of 10 to 25 Hz for 30-minute sessions. VBP was measured using laser Doppler flowmetry, focusing on neurogenic oscillations. The results revealed that 75.8% of the stimulation trials led to significant increases (≥500%) in neurogenic blood flow energy, most commonly observed between 20 to 35 minutes after stimulation onset. These findings suggest that prolonged stimulation durations are critical for achieving significant responses. Interestingly, the effects on blood perfusion were independent of stimulation frequency or estrous cycle phase, indicating a consistent neurogenic response. This research supports the potential of TNS to enhance pelvic blood flow, which could directly improve genital arousal, lubrication, and orgasm in women with SD [41].

TNS was investigated for its immediate and long-term effects on sexual motivation and receptivity in female rats. The study utilized two experiments involving ovariectomized rats exposed to various treatment conditions combining TNS with hormone priming. Behavioral outcomes were evaluated using an operant chamber setup, where access to a sexually active male was controlled through nose pokes. In the first experiment, rats rotated through different conditions weekly for 10 weeks, and behavioral metrics were assessed immediately after stimulation. Results showed non-significant trends of increased sexual motivation, as indicated by nose poking activity, but no significant changes in receptivity metrics such as lordosis quotient or mounts. In the second experiment, rats underwent a single treatment condition for six weeks, with behavior tracked longitudinally. While immediate effects on sexual motivation remained subtle, long-term TNS combined with hormone priming exhibited trends of increased sexual receptivity over time, particularly in metrics related to interaction with the male [42].

These findings suggest that TNS may enhance sexual receptivity when applied consistently over an extended period. Though statistical significance was not achieved, likely due to small sample sizes, the results underscore the potential of TNS as a neuromodulation therapy for FSD, warranting further exploration with larger cohorts.

Advancing FSD management in MS: the emerging role of TTNS 

TTNS and FSD were evaluated through several clinical pilot studies. A study examined the effects of weekly nerve stimulation sessions on sexual function in women with FSD over 12 weeks. Participants included women experiencing challenges in arousal, lubrication, and orgasm. Researchers utilized validated measures such as the Female Sexual Function Index (FSFI) to assess outcomes. Participants reported significant improvements across FSFI domains, particularly in lubrication, arousal, and orgasm, which are closely associated with physiological aspects of sexual function. These results align with preclinical findings indicating that TNS enhances genital blood flow, suggesting a potential mechanism for its efficacy. Long-term effects were particularly notable, with sustained benefits observed six weeks after the conclusion of the intervention. This study demonstrates that consistent, repeated neuromodulation may be more effective than on-demand approaches for addressing FSD [43]. 

A pilot randomized control trial examined the efficacy of TTNS in addressing primary SD in patients with MS. The study included 40 participants randomized into TTNS and sham groups, receiving three weekly sessions over two months. Pre- and post-intervention evaluations using the Multiple Sclerosis Intimacy and Sexuality Questionnaire (MSISQ-15) revealed statistically significant reductions in SD severity in the TTNS group. These findings underscore the neuromodulation potential of TTNS in enhancing sexual function by targeting neural pathways shared by the bladder and sexual organs. Improvements in bladder-related symptoms further suggest overlapping neurophysiological mechanisms. TTNS demonstrated significant improvements in erectile function (males), vaginal lubrication (females), orgasm quality, satisfaction, bladder-related symptoms, and sexual desire compared to the sham group. The study highlights TTNS as a non-invasive, safe, and effective therapeutic option for MS-related SD. However, limitations such as a small sample size and reliance on self-reported outcomes warrant further research [44]. 

TTNS in combination with pelvic floor muscle training (PFMT) is an effective treatment for the lower urinary tract symptoms in women with MS. A RCT included three groups of patients: one group underwent PFMT with sham neuromuscular electrical stimulation (NMES), another combined PFMT with intravaginal NMES, and the third group combined PFMT with TTNS. All treatment methods significantly improved LUTS symptoms, including reductions in urgency and urinary incontinence episodes, and improved pelvic floor muscle (PFM) strength, flexibility, and relaxation [45]. 

Lucio et al. evaluated the impact of PFMT alone or in combination with NMES or TTNS on SD in women with MS. Thirty participants with relapsing-remitting MS and SD were randomized into three groups: PFMT with electromyographic (EMG) biofeedback and sham NMES (Group I), PFMT with EMG biofeedback and intravaginal NMES (Group II), and PFMT with EMG biofeedback and TTNS (Group III). The findings revealed significant improvements across all groups in PFM function. Notably, Group II achieved the most substantial improvements in PFM tone, vaginal flexibility, and relaxation post-contraction. FSFI scores demonstrated improvements in arousal, lubrication, satisfaction, and total sexual function across all groups [46]. 

Polat Dunya et al. compared the impact of two non-invasive treatments, TTNS and PFMT, on SD in women with MS who also report OAB symptoms. Participants were divided into TTNS and PFMT groups. Assessments were conducted at baseline and after six weeks using the FSFI, Overactive Bladder Questionnaire (OAB-v8), and Sexual Quality of Life-Female (SQoL-F) questionnaire. Results revealed significant improvements in FSFI scores in both groups, with TTNS showing enhancements in all domains except pain, and PFMT improving desire, orgasm, and satisfaction. OAB symptoms and sexual quality of life also improved in both groups. Notably, there were no significant differences between the two methods in terms of overall efficacy. However, there are limitations such as the lack of a control group and the short follow-up periods [31]. Another study conducted by the same research group, involving a larger patient cohort, has confirmed the findings of the initial study [40]. 

Short-term TTNS was evaluated as a treatment for FSD in women with SCI. Studies showed that TNS had limited efficacy in improving physiological markers of genital arousal, such as vaginal blood flow. Unlike pudendal nerve stimulation, which demonstrated significant increases in genital perfusion, TNS produced minimal changes in blood perfusion in both spinally intact and spinalized animal models. Furthermore, subjective arousal and genital sensations were inconsistently reported in human trials involving TNS, with no significant improvement observed in sexual function metrics for SCI participants [47].

These findings suggest that TNS may not effectively engage the neural pathways required to modulate genital arousal in women with SCI. Its reliance on intact supraspinal pathways may limit its utility for individuals with complete SCI [48]. However, some SCI participants expressed interest in continuing neuromodulation treatments, indicating potential non-physiological benefits or a willingness to explore further research. This highlights the importance of developing targeted neuromodulation approaches tailored to the unique neurological profiles of women with SCI and understanding the mechanisms behind their effectiveness in treating FSD.

A pilot study investigated the effects of TTNS on ED among elderly patients. TTNS sessions were administered three times a week, employing electrodes to stimulate the posterior tibial nerve. Efficacy was measured using the Arizona Sexual Experience Scale (ASEX). Results indicated a significant improvement in erectile function, with median ASEX scores decreasing from 24 (pre-treatment) to 18 (post-treatment). Statistical analysis confirmed the changes were not due to chance (p<0.001). The study highlights TPTNS as a non-invasive, safe, and effective approach to managing ED. By stimulating sacral nerve fibers, TPTNS may enhance neurological pathways involved in erectile function, improving overall sexual health and quality of life in elderly individuals [49]. 

## Conclusions

Emerging evidence supports the role of TTNS as a safe, non-invasive, and effective therapeutic option for FSD in women with MS. Clinical studies indicate significant improvements in domains such as arousal, lubrication, and orgasm, potentially linked to enhanced pelvic perfusion and neuromodulation of shared bladder and sexual pathways. When combined with PFMT, TTNS appears to further enhance pelvic floor strength, alleviate overactive bladder symptoms, and improve overall sexual quality of life.

Further large-scale, long-term studies with standardized protocols are needed to confirm these promising findings, clarify underlying mechanisms, and optimize clinical applications. Additionally, targeted neuromodulation strategies may be required for women with complete SCI. Overall, TTNS represents a promising addition to the multidisciplinary management of FSD in MS, warranting continued research and integration into clinical practice.
